# Case report: Invasive neuromonitoring in status epilepticus induced hypoxic ischemic brain injury

**DOI:** 10.3389/fneur.2023.1284098

**Published:** 2023-11-30

**Authors:** Karandeep Singh Bhatti, Swarna Rajagopalan

**Affiliations:** Neurological Institute, Cooper University Hospital, Camden, NJ, United States

**Keywords:** invasive neuromonitoring, diffuse cerebral edema, continuous EEG monitoring, bilateral decompressive craniectomy, refractory intracranial hypertension, ICP monitoring, refractory status epilepctius, brain tissue oxygen monitoring

## Abstract

**Objectives:**

Literature on invasive neuromonitoring and bilateral decompressive craniectomies (BDC) in patients with refractory status epilepticus (RSE)-mediated hypoxic-ischemic brain injury (HIBI) is limited. Neuromonitoring can guide decision making and treatment escalation.

**Methods and results:**

We report a case of a 17 years-old male who was admitted to our hospital’s intensive care unit for RSE. HIBI was detected on neuroimaging on this patient’s second day of admission after he developed central diabetes insipidus (DI). Invasive neuromonitoring revealed raised intracranial pressure (ICP) and brain hypoxia as measured by reduced brain tissue oxygen tension (PbtO_2_). Treatments were escalated in a tiered fashion, including administration of hyperosmolar agents, analgesics, sedatives, and a neuromuscular blocking drug. Eventually, BDC was performed as a salvage therapy as a means of controlling refractory ICP crisis in the setting of diffuse cerebral edema (DCE) following HIBI.

**Discussion:**

SE-mediated HIBI can result in refractory ICP crisis. Neuromonitoring can help identify secondary brain injury (SBI), guide treatment strategies, including surgical interventions, and may lead to better outcomes.

## Introduction

**Figure 1:**
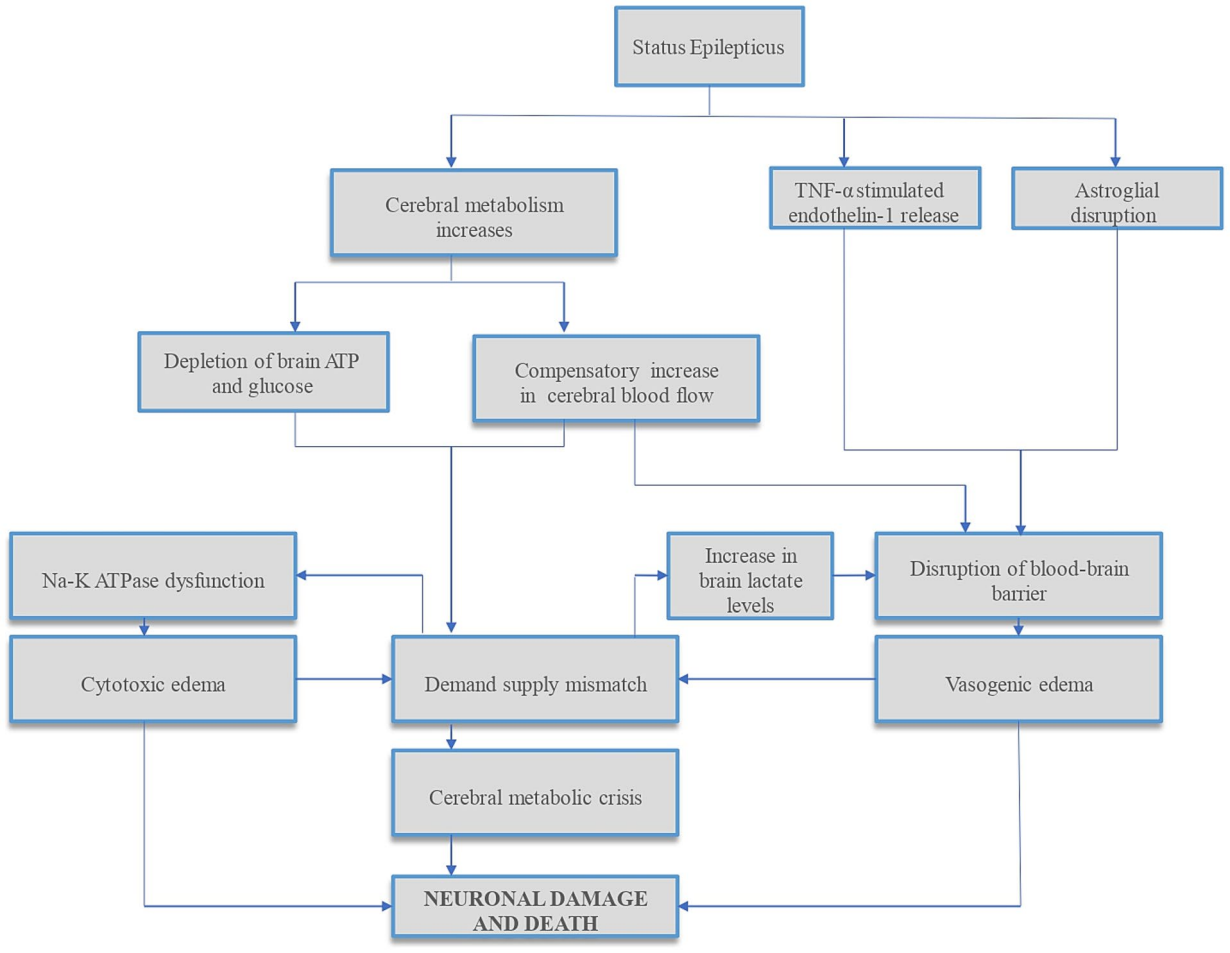
Summary of mechanisms of status epilepticus-induced neuronal injury. Status epilepticus (SE) results in disrupted glucose and oxygen delivery and/or utilization, reducing brain adenosine triphosphate (ATP) and increasing cerebral lactate levels. In addition, the increased metabolic demands during SE result in further ATP depletion, leading to sodium–potassium (Na–K) ATPase pump dysfunction and cytotoxic edema. To compensate for increased cerebral metabolic demands, cerebral blood flow (CBF) increases. This CBF increase, especially in the dysregulated brain, can cause hyperperfusion-induced blood-brain barrier (BBB) disruption and vasogenic edema, further increasing ICP. In addition, studies have found complex molecular mechanisms of SE-induced BBB dysfunction via tumor necrosis factor-alpha (TNF-α) stimulated endothelin-1 release (6) and dystrophin/α-syntrophin complex-mediated astroglial aquaporin 4 (AQP4) disruptions (7) which can compound cerebral edema and raise ICP. Both cytotoxic and vasogenic edemas can further increase the demand-supply mismatch. This cascade of events can lead to cerebral metabolic crisis and neuronal cell death. ATP, adenosine triphosphate; Na–K, sodium–potassium; TNF-α, tumor necrosis factor-alpha.

Refractory status epilepticus (RSE) can rarely result in diffuse cerebral edema (DCE), which is often fatal (1) when it occurs. Increased metabolic demand of the brain along with reduced substrate delivery to the brain or inability to effectively utilize glucose and oxygen can deplete brain adenosine triphosphate (ATP), increase cerebral lactate levels, and decrease pH in the brain interstitium. Impaired cerebral autoregulation, and concomitant systemic factors such as hypotension, hypoxia from aspiration, respiratory acidosis from apnea and metabolic acidosis from seizures can compound neuronal damage that occurs in status epilepticus (SE) (2–4). Compensatory increased cerebral blood flow (CBF), especially in dysregulated brain can result in hyperperfusion-induced blood-brain barrier (BBB) disruption and worsen vasogenic edema (5). Studies have found complex molecular mechanisms of SE-induced BBB dysfunction (6, 7), summarized in Figure 1, along with other mechanisms of neuronal injury in SE.

Intracranial hypertension, metabolic crisis and hypoxic-ischemic brain injury (HIBI) have been described in SE patients. However, literature is primarily focused on SE that occurs following other primary forms of acute brain injury (ABI) such as intraparenchymal hematoma, cardiac arrest, subarachnoid hemorrhage (SAH), or traumatic brain injury (TBI), which in itself may impact pathophysiology of RSE (8–11). We found limited literature on development of DCE (1) in patients with SE without other primary forms of ABI, and no literature on the use of invasive neuromonitoring (INM) to guide clinical care of these patients. We present a case report of a 17 years-old man with a history of generalized epilepsy who developed intractable intracranial hypertension secondary to RSE-induced cerebral edema and HIBI, underwent INM, cerebral physiology-targeted interventions, and ultimately bilateral decompressive craniectomies (BDC).

## Case description

A 17 years-old man with a past medical history of idiopathic generalized epilepsy was brought to our tertiary care hospital after he was witnessed to become unresponsive with a blank stare. Upon arrival at the emergency room (ER), the patient was lethargic, opened eyes to minor tactile stimuli, blinked to threat, and withdrew all extremities to painful peripheral stimuli; however, he failed to follow commands or produce spontaneous speech. He was febrile with a *T*_max_ of 101.5 F, and although his total leukocyte count was normal, he was initiated on empiric antimicrobials. The patient’s point of care glucose was 111 and his pulse oximetry reflected an oxygen saturation of 100%. The patient’s fever rose suspicion for meningoencephalitis causing breakthrough seizures. Chest X ray, urinalysis, and blood cultures were included in to broaden the infectious work-up. Urine drug screen was not conducted, however the patient’s family strongly denied a history of illicit drug use in the patient. The initial non-contrast head CT (NCHCT) did not reveal an acute intracranial pathology. Thereafter, the patient had three back-to-back generalized tonic-clonic seizures without returning to baseline; thus, he was given intravenous (IV) loads of lorazepam and levetiracetam. Subsequently, another clinical seizure ensued during which he was loaded with IV midazolam, intubated for airway protection, and transferred to the intensive care unit (ICU).

**Figure 2:**
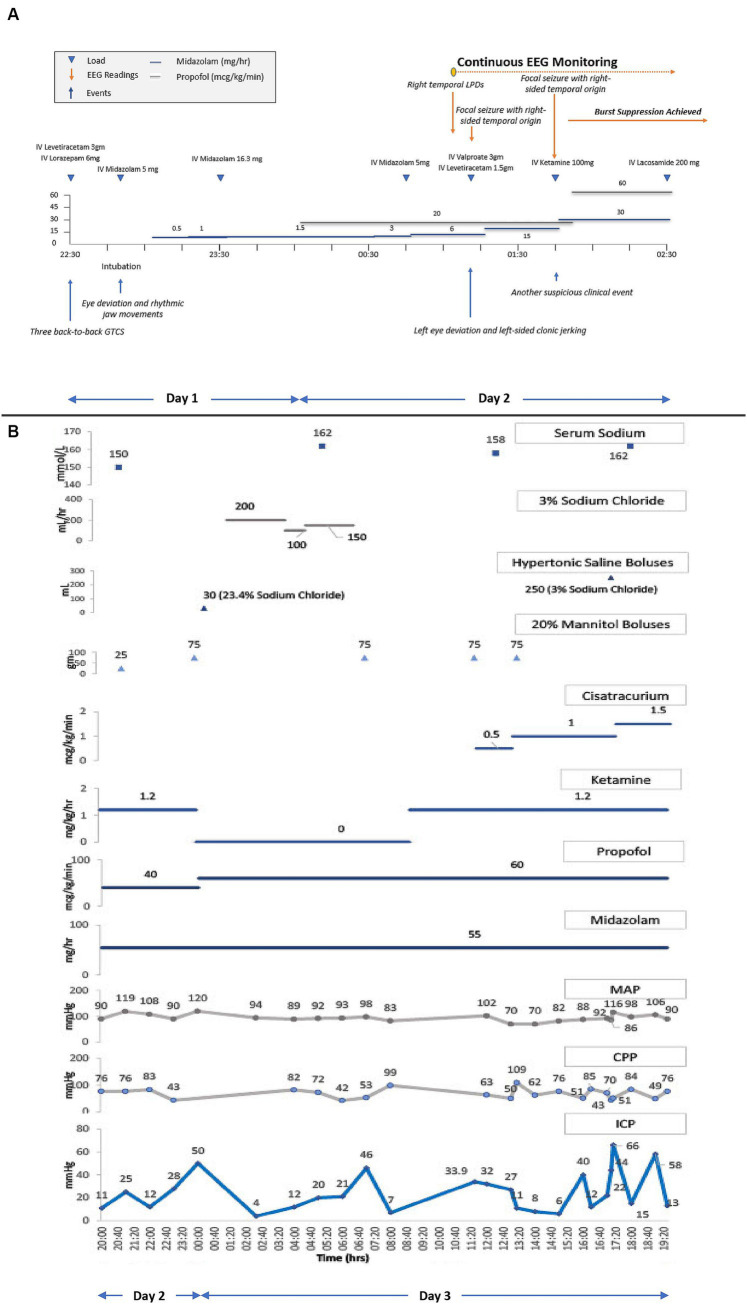
**(A)** Refractory status epilepticus treatment timeline. cEEG, continuous electroencephalography. Shows a detailed timeline from the patient’s first seizure to the initiation of cEEG monitoring to burst-suppression. Triangles connotate boluses or loads, and lines connotate continuous infusions. The patient received loads of IV levetiracetam, IV lorazepam, and IV midazolam, and underwent endotracheal intubation for airway protection. Following intubation, IV midazolam and IV propofol infusions were initiated and up-titrated for suspicion of ongoing non-convulsive status epilepticus (NCSE). Upon initiation of cEEG monitoring, right temporal lateralized periodic discharges (LPDs) and right temporal focal seizures were seen, prompting an increase in the rate of midazolam infusion with additional IV loads of valproate, levetiracetam, and ketamine, to achieve burst suppression. **(B)** ICP, CPP, MAP, and serum sodium trends along with the titration of sedatives, hyperosmolar therapies, and neuromuscular blocker infusions. ICP, intracranial pressure; CPP, cerebral perfusion pressure; MAP, mean arterial pressure. Triangles connotate boluses, and lines connotate continuous infusions. ICP monitoring was initiated on the second day of hospitalization after the onset of clinical DI and radiographic diffuse cerebral edema. Propofol, versed, and ketamine infusions were already running to maintain burst suppression for RSE. The initial ICP readings between 25–28 mmHg were treated with a mannitol bolus. A subsequent ICP peak of 50 mmHg was treated with hyperventilation, an increase in the propofol infusion rate, a mannitol and hypertonic saline bolus, followed by a hypertonic saline infusion. Ketamine infusion was discontinued due to a concern of possibly contributing to intracranial hypertension, but after multidisciplinary rounds on the next day, it was restarted. Shortly thereafter, the patient’s ICP went up to 46 mmHg, for which the patient received another mannitol bolus which resulted in a transient ICP reduction before another ICP crisis to 32–34 mmHg. These subsequent ICP surges were treated with two mannitol boluses in conjunction with the initiation and rapid up-titration of cisatracurium infusion. ICP was transiently reduced, but became sustained at 40–70 mm Hg, despite an increase in the rate of cisatracurium, a hypertonic saline bolus, and continuation of all previous therapies.

Propofol and midazolam infusions were initiated empirically while awaiting continuous electroencephalography (cEEG) monitoring initiation. In the interim, the patient received two additional midazolam boluses in suspicion of ongoing non-convulsive status epilepticus (NCSE). Upon initiation of cEEG, the patient was found to have right-sided temporal lateralized periodic discharges (LPDs) followed by two clinical seizures. Serial escalation with IV anti-seizure medications (ASMs) and IV sedatives including valproate, levetiracetam, and ketamine resulted in a burst-suppression pattern on cEEG. Figure 2A depicts a detailed timeline from the patient’s first seizure to the initiation of cEEG monitoring to burst-suppression.

The following afternoon, the patient developed polyuria, and serum and urine chemistries were consistent with new-onset diabetes insipidus (DI). Due to clinical suspicion of a central cause of DI, an urgent NCHCT was performed. It showed an acute diffuse loss of grey-white differentiation, consistent with DCE, a new finding compared to his initial imaging. The patient had not suffered any interval episodes of systemic hypoglycemia or hypoxia, and the patient remained afebrile. A CT venogram demonstrated patent venous outflow. A lumbar puncture was performed and yielded clear cerebrospinal fluid (CSF) with an opening pressure of 30 mmHg, nucleated cell count of 2/μL, red cell count of 148/μL, glucose of 90 mg/dL, and protein of 181 mg/dL. Infectious CSF labs were sent including bacterial/fungal cultures, and herpes simplex virus (HSV) 1/2 PCR. Since the patient’s clinical presentation, with the exception of SE, were not suggestive of viral encephalitides, additional viral studies were not sent.

After a multidisciplinary discussion between the neurointensivist and the neurosurgeon, a decision was made to pursue INM to institute treatments that may reduce secondary brain injury (SBI) in a timely way. The neurosurgery team placed an intraparenchymal (IP) monitor (Raumedic Neurovent-PTO) in the right frontal lobe, consisting of an intracranial pressure (ICP) and brain tissue oxygen tension (PbtO_2_) monitor. After the initial calibration period, PbtO_2_ readings were 3–7 mmHg, reflecting a severely hypoxic brain. Six hours following placement of IP monitor, the ICP peaked to 50 mmHg. This was transiently abated by hyperventilation, an increase in IV propofol rate, and additional boluses of IV 20% mannitol and 23.4% sodium chloride. Treatment with hyperosmolar therapy with concomitant DI resulted in hypernatremia. Hypernatremia was managed by one-to-one replacement of urine fluid losses with isotonic fluids. Hypotonic fluids were not used to prevent overcorrection of sodium, especially in the presence of DCE and raised ICP. Clinical DI was transient, and treated with 1mcg of desmopressin. The next morning, the patient underwent a non-contrast MRI brain which showed diffuse grey matter FLAIR and DWI hyperintensities with ADC pseudonormalization reflective of edema. A slight improvement in grey-white differentiation was appreciated on this MRI in comparison to the prior NCHCT. Despite this slight radiographic improvement, the IP monitor continued to reflect persistent ICP crisis ranging between 30–60 mmHg refractory to all medical therapies, including sedatives, analgesics, hyperosmolar therapies, and neuromuscular blocking drug administration. Detailed pharmacological treatments are shown graphically, along with physiological trends of ICP, mean arterial pressure (MAP), and cerebral perfusion pressure (CPP) in Figure 2B.

Given refractory ICP crisis, following a multidisciplinary discussion, the patient underwent BDC, removal of the IP monitor and an external ventricular drain (EVD) placement. Following BDC, the patient’s ICP crisis resolved and ICPs remained within the normal range. Clinical DI did not recur, and hypernatremia was attributed primarily to hyperosmolar therapy. After normal readings of ICP for 24 h, the EVD was removed, and the sedatives were weaned off. While the initial indication of burst suppression in our case was RSE, it was continued for treatment of intracranial hypertension, for a total period of 72 h. All the infectious work up, including CSF studies, resulted negative in a few days, and antimicrobials were discontinued. In the absence of pleocytosis, elevated protein in the CSF was considered to reflect non-specific neuronal inflammation in the setting of RSE and HIBI. Fever at initial presentation was attributed to RSE and being found unresponsive outside under a high-temperature weather.

**Figure 3:**
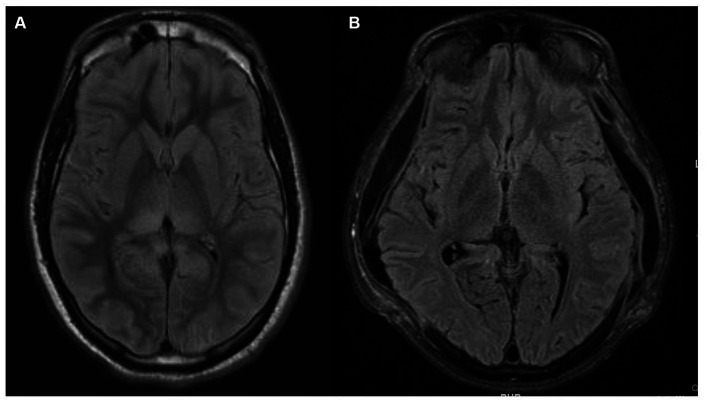
Magnetic resonance imaging (MRI) brain fluid-attenuated inversion recovery (FLAIR) sequences. **(A)** MRI brain FLAIR sequence that demonstrates bright T2/FLAIR signal, suggesting diffuse grey matter edema which could indicate hypoxic-ischemic brain injury. **(B)** Repeat MRI brain FLAIR sequence a month later, showing near complete resolution of prior abnormalities and expected extra-cranial herniation of brain through the craniectomy sites.

The patient’s ICU length of stay, complicated by aspiration pneumonia, followed by acute respiratory distress syndrome requiring venovenous (VV) extracorporeal membrane oxygenation (ECMO), was 30 days. Following extubation, 24 days from admission, the patient aroused to verbal stimuli, visually tracked people, and began following simple axial and appendicular commands. Over the next few days, his neurologic exam continued to improve as he was more persistently awake and began to respond in one to two words. At the time of discharge, after a total hospital stay of 50 days, the patient was awake, alert, oriented, mildly inattentive, produced spontaneous fluent speech, followed simple and complex commands, without any detectable cranial nerve, motor, or sensory abnormalities. A repeat MRI brain almost a month after the first MRI showed complete resolution of the previously noted radiographic abnormalities (Figure 3).

## Discussion

To the best of our knowledge, this is the first case report using INM to guide management in refractory intracranial hypertension in a patient with SE-induced diffuse cerebral edema, in the absence of other primary ABI, and to survive it with a good neurologic outcome. In the setting of SE following ABI due to other primary causes such as intraparenchymal hemorrhage, cardiac arrest, SAH, and TBI, we found case reports of the use of INM showing brain hypoxia, increased ICP (8, 9, 11), hyperemia (9, 10), and cerebral metabolic crisis (9, 11). While these studies demonstrate the effects of seizures on cerebral physiology, these patients had concomitant primary ABI as a cause of seizures, therefore many of the physiological changes were attributable to their primary ABI rather than effects of seizures alone.

Sedatives were titrated to achieve burst suppression to treat RSE, as supported per guidelines (12), however optimal intensity and duration of sedation, as well as goals of seizure or burst suppression remain to be determined. A recent study showed that suppressing rhythmic and periodic EEG activity for at least 48 h in HIBI patients did not alter neurological outcome (13), however this cannot be extrapolated to other types of ABI, including secondary HIBI from RSE rather than cardiac arrest. Addition of ketamine to propofol and midazolam infusions have shown reduced seizure burden and increased seizure termination in RSE and super refractory status epilepticus (SRSE) in both adult and pediatric populations (14, 15).

New-onset DI raised the clinical suspicion of intracranial hypertension. Prompt institution of INM confirmed intracranial hypertension and brain hypoxia, enabling timely escalation of medical treatments and, ultimately, BDC, preventing transtentorial herniation and death.

Hyperosmolar therapy was instituted to reduce ICP. Mannitol and hypertonic saline reduce ICP by way of fluid shift from intracellular to the extracellular space. In addition, mannitol promotes osmotic diuresis and alters blood viscosity (16). Sedatives and analgesics were initially used to treat seizures, pain and anxiety, but these doses were titrated up to reduce the cerebral metabolic rate of oxygen (CMRO_2_) during periods of intracranial hypertension. Reduction in CMRO_2_ reduces the cerebral metabolic demand in the setting of demand-supply mismatch during SE, which is crucial in preventing further neuronal damage during periods of metabolic crisis. The reduction in CMRO_2_ also leads to concurrent decreases in CBF, cerebral blood volume, and ultimately ICP (17, 18). In this case, ketamine was continued alongside propofol and midazolam for sedation, based on recent studies dispelling concerns of elevated ICP associated with ketamine use (19). Notably, a retrospective study in the pediatric severe TBI population suggested that ketamine might aid in reducing ICP during ICP crisis (20). A MAP augmentation challenge was performed to identify if ICP crisis was perfusion-limiting, i.e., oligemic or perfusion-driven, i.e., hyperemic and suggested a mixed picture, typical of heterogenous injury (21). As ICPs remained refractory of treatment with sedatives and hyperosmolar therapy, cisatracurium, a neuromuscular blocking agent (NBMA), was introduced to further reduce CMRO_2_ by promoting ventricular synchrony and preventing ICP peaks from coughing, suctioning, motion, pain, or shivering (22), however, there is a dose-dependent association with increased neuromuscular weakness and morbidity (23).

IP monitor removal during BDC was followed by an EVD placement for continued ICP monitoring and to allow therapeutic lowering of ICP via CSF diversion, which may have helped to reduce ICP further. The two most commonly used techniques to measure ICP, with good correlation, are the fluid-coupled EVD and strain-gauge or fiberoptic-based IP monitors. One may be preferentially chosen based on the need for CSF drainage, interest in regional cerebral physiology, need for continuous ICP measurements or feasibility. In this case, the decision to place an EVD was based on both the option of therapeutic ICP lowering and clinical feasibility (24).

**Figure 4:**
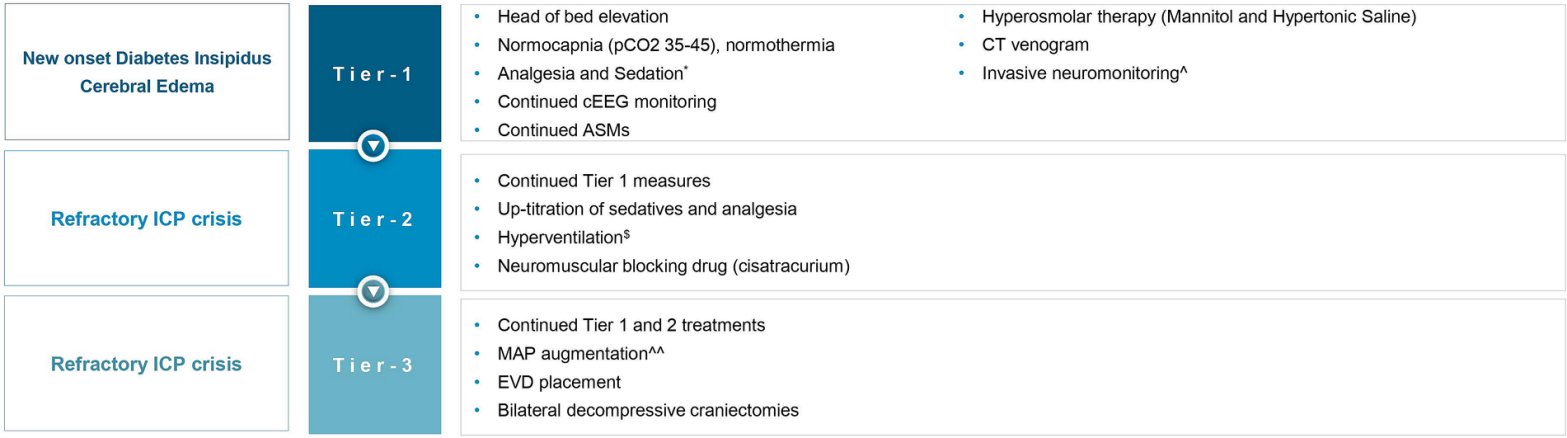
Invasive neuromonitoring-guided treatment for refractory intracranial hypertension and brain hypoxia. ICP, intracranial pressure; pCO_2_, partial pressure of carbon dioxide; cEEG, continuous electroencephalography; ASMs, anti-seizure medications; MAP, mean arterial pressure; EVD, external ventricular drain. ^*^The patient was already on propofol, midazolam, and ketamine, before the onset of global hypoxic brain injury, for status epilepticus, now up-titrated to treat ICP crisis. ^$^Briefly targeting hypocapnia. ^^^Using an intraparenchymal monitor (Raumedic Neurovent-PTO). ^^^^To identify if ICP elevation was perfusion-limiting (oligemic) or driven (hyperemic).

Although literature explores the use of BDC in TBI (25), SAH (26) and cerebral venous sinus thrombosis (27), we found no literature on BDC for DCE and medically refractory intracranial hypertension in HIBI, and particularly SE-induced HIBI. In this report, we describe the first case report of a patient where BDC was utilized as a means of controlling refractory elevated ICP in the setting of DCE following SE-induced HIBI. The rationale for instituting BDC as a salvage therapy is rooted in Monroe Kellie doctrine, as the removal of the “rigid” cranial vault allows cerebral expansion extracranially, resulting in reduced ICP and improved cerebral perfusion. Pentobarbital coma was not offered as the patient was already in burst suppression with the other management strategies, and given the diffuse nature of cerebral edema, increasing the intensity of burst suppression further was not expected to address the intracranial hypertension and brain hypoxia further. A multidisciplinary consensus was achieved that maximal intensity of medical therapies was offered in this case prior to the decision of pursuing BDC. Unilateral decompressive hemicraniectomy (DHC) was felt to be an inadequate therapeutic option given the diffuse nature of cerebral edema demonstrated on neuroimaging. Given paucity of data in this particular clinical situation, a detailed and informed conversation explaining risks and benefits was conducted with the patient’s family, who elected to proceed with BDC. Our physiology-based algorithm using INM (Figure 4) data, drove a tiered approach for refractory intracranial hypertension and brain hypoxia in the setting of severe cerebral edema and HIBI following SE. Based on radiographic cerebral edema alone without INM, it is unlikely that this intensity of treatments would have been offered, and death from cerebral herniation may have been a realistic outcome. In this case, treatment was escalated to the highest tier, requiring BDC as a salvage therapy, which was effective at both reducing ICP immediately, and preventing death. Though ultimately whether treatments modified the course of SBI is unknown, we believe the tiered medical management limiting prolonged periods of intracranial hypertension and brain hypoxia, and expeditious decision to proceed with BDC once it was evident that intracranial hypertension was becoming refractory to medical therapies not only prevented this patient’s death, but may have also limited neuronal loss and contributed to the patient’s excellent eventual neurologic recovery.

Limitations of this report include the loss of continuous data capture, especially PbtO_2_ recordings due to manual data entry into the electronic medical record, given the retrospective nature of this case report. Data loss also limited measurement of markers of cerebral autoregulation, such as cerebral pressure and oxygen reactivity indices. Our intensive care unit is not equipped to perform cerebral microdialysis, so while this data may have added further information, this was not performed.

In conclusion, our case report implores intensivists to consider the use of invasive neuromonitoring to guide management in patients who suffer HIBI secondary to SE and to consider BDC as a salvage treatment option in cases with refractory intracranial hypertension and brain hypoxia.

## Data availability statement

The original contributions presented in the study are included in the article/supplementary material, further inquiries can be directed to the corresponding author.

## Ethics statement

Ethical review and approval was not required for the study on human participants in accordance with the local legislation and institutional requirements. Written informed consent to participate in this study was provided by the patient’s legal guardian/next of kin. Written informed consent was obtained from the participant/patient(s) legal guardian/next of kin for the publication of this case report.

## Author contributions

KB: Data curation, Writing – original draft. SR: Conceptualization, Writing – review & editing.
